# Prevalence and associated factors of common mental disorders among adult patients attending HIV follow up service in Harar town, Eastern Ethiopia: a cross-sectional study

**DOI:** 10.1186/s40359-019-0281-4

**Published:** 2019-02-22

**Authors:** Aboma Motumma, Lemma Negesa, Gari Hunduma, Tilahun Abdeta

**Affiliations:** 1 Department of Nursing, College of health and medical sciences, School of Nursing and Midwifery, Haramaya University, Harar, Ethiopia; 2 Department of Psychiatry, College of health and medical sciences, School of Nursing and Midwifery, Haramaya University, Harar, Ethiopia; 3 Centre for International Health, Ludwig Maxmillians University, Munich, Germany

**Keywords:** Prevalence, Associated factors, CMDs, HIV/AIDS, Adult patients, Ethiopia

## Abstract

**Background:**

In developed countries, there are well documented mental health impacts of HIV/AIDS and patients’ quality of life. Acquiring HIV/AIDS can be a serious psychological trauma and can predispose a person to different mental disorders. Co-occurring mental illness complicates diagnosis, help-seeking, quality of care provided, treatment outcomes and adherence. However, in Ethiopia, studies about mental health problems in HIV/AIDS treatment settings are limited. The purpose of the current study is to determine the prevalence and associated factors of common mental disorders among adult HIV/AIDS patients undergoing HIV service in Harar town, eastern Ethiopia. Conducting this study is important as baseline information for the concerned stakeholders including health professionals and policymakers and in general to improve the quality of care for HIV/AID patients.

**Methods:**

Institution based cross-sectional study was conducted. We collected data from 420 adult patients through a face to face interviewing technique using a standardized questionnaire and review of medical records. Bivariable and multivariable (binary logistic regression) analyses were used to check the association between common mental disorders (CMDs) and independent variables. Variables which have a *p*-value < 0.05 during bivariable regression were entered into multivariable (binary logistic regression) and finally which have a p-value of < 0.05 under multivariable (binary logistic regression) were identified as statistically significant association at 95% of confidence interval.

**Results:**

All 420 patients were interviewed providing response rate 100%. The result revealed that (28.1%; 95% CI; 26.14, 30.06) of HIV/AIDS patients had CMD. In the final model, stage 4 HIV/AIDS (Adjusted Odds Ratio 3.37, 95% CI: 1.45, 7.83), family history of mental illness (AOR 2.65, 95% CI: 1.26, 5.54) and current drinking alcohol (AOR 5.1, 95% CI: 2.04, 12.79) were found having statistically significant association with CMD.

**Conclusions:**

This study investigated the prevalence and associated factors of CMD among adults living with HIV/AIDS. HIV/AIDS stage, having family history of mental illness and current drinking alcohol were the main identified associated factors of CMD. These factors are important for the hospitals and other concerned bodies for providing prevention and appropriate intervention of common mental disorders among HIV/AIDS patients.

## Background

Mental illness is among many non-AIDS complications that limit the HIV/AIDS patients’ quality of life (QOL) [1, 2]. Mental illness and HIV infection are linked in many ways including (*I*) HIV infection often result in serious emotional disturbance due to its malignant course and associated stigma [3]. (*II*) HIV has direct effects on a central nervous system which may lead to cognitive, perceptual and behavioral changes [3]. (*III*) Mental illness can be a consequence of opportunistic neurological and systemic infections and their treatments [4]. (*IV*) Some Highly Active Antiretroviral Therapy (HAART) have psychiatric side effects [4]. (*V*) Patients of severe mental illness are at risk to HIV infection [5]. (*VI*) HIV and psycho-active Substance use is connected in direct ways (IV use) and in indirect ways influencing sexual behavior [3–5]. There are many reported impacts of mental health problems among HIV/AIDS patients including speeding up the disease progression, reducing adherence to treatments, affecting willingness or ability to access health care and increased risk of transmission of other sexually transmitted infections (STI) by increasing high-risk behaviors [6].

Common mental disorders (CMDs) is set of signs and symptoms of non-psychotic depression, anxiety, and somatoform disorders and it is frequently reported among HIV infected people [7–12]. The magnitude of major depressive disorder (MDD) ranges from 16.2 to 36% among HIV patients in the USA [13]. This is as compared to the general population’s prevalence 4.2%, it is 4 to 7 fold greater [14]. Another study in LAMIC (Kenya, Democratic Republic of Congo and Thailand) again reported that depression is higher in HIV patients than in HIV negative individuals [15]. However, these studies on co-occurring of mental illness and HIV-infection is still limited in sub-Saharan Africa including Ethiopia [16–18]. The study on Anxiety Disorders among adult HIV/AIDS patients in a Sub-Saharan Africa revealed that the magnitude of anxiety disorders and mixed anxiety-depressive disorder among adult PLWHA was 21.7, and 5.3% respectively. Lack of family support, unemployment and being unmarried were factors significantly associated with anxiety disorders among participants [19]. Study in Nigeria showed that individuals with HIV had significantly higher rates of common mental disorder (OR = 3.58, 95% CI = 1.44–8.94) than healthy people and stage of the HIV was significantly associated with common mental disorder [7]. A systematic review and meta-analysis result in sub-Saharan Africa revealed that prevalence estimates of depression ranged between 9 and 32% in PLWHA on Antiretroviral Therapy (ART) and in untreated or mixed (treated/untreated) ones. Low socio-economic conditions in PLWHA on ART, female sex and immunosuppression in mixed/untreated PLWHA were reported associated factors [20].

The aim of the current study is to determine the magnitude of common mental disorders and factors associated with it among HIV positive individuals undergoing HIV services in Harar town, Eastern Ethiopia. It is important as baseline information for the concerned stakeholders including health professionals and policymakers and in general to improve the quality of care for HIV/AID patients.

## Methods

### Study Area

We conducted the study in Harar town, Harari regional state, Eastern Ethiopia, at Hiwot Fana specialized University and Jugel governmental Hospital. Location of Harar is 527 km from the capital city of Ethiopia, Addis Ababa to the direction of the east. In Harar town, there are 19 health posts, 2 private hospitals, 8 health centers, 1 FGAE (Family Guidance Association of Ethiopia) clinic, 3 government hospitals and one university specialized hospital. During our study, a total of 7558 HIV patients were enrolled at ART clinic in the town. Hiwot Fana and Jugel hospitals are the major ART sites in the region.

### Study design

We conducted through facility-based Cross-sectional study design.

### Source population

Adult Sero-positive individuals in Harar town, Eastern Ethiopia.

### Study population

The 420 randomly selected adult HIV patients enrolled in HIV services (Pre-ART and on ART) at Hiwot Fana specialized University Hospital and Jugel governmental Hospital in Harar town, Eastern Ethiopia and who were 18 years or older were included in the study and patients who were critically ill during data collection period were excluded.

### Sample size determination

We calculated Sample size using single population proportion formula taking *p* = 46.7% from previous study “Prevalence of common mental disorders among HIV/AIDS patients in Ethiopia” [8], d of precision 5, 95% confidence interval and 10% non- response rate.

$$ {\displaystyle \begin{array}{c}\mathrm{n}=\frac{{\left(\mathrm{z}\;\upalpha /2\right)}^2\mathrm{p}\left(1-\mathrm{p}\right)}{{\mathrm{d}}^2}\kern1em \mathrm{where}\kern1em \mathrm{n}=\mathrm{sample}\kern0.5em \mathrm{size},\kern0.5em \mathrm{Z}\upalpha /2=\mathrm{Z}\kern0.5em \mathrm{score}\kern0.5em \mathrm{at}\kern0.5em 95\%\kern0.5em \mathrm{CI}=1.96,\kern0.5em \mathrm{p}=46.7\%\kern0.5em \mathrm{and}\kern0.5em \mathrm{d}=5\%\kern0.5em (0.05)\\ {}\mathrm{n}=\frac{(1.96)^20.467\left(1-0.467\right)}{(0.05)^2}=\frac{3.8416\times 0.467\times 0.533}{0.0025}=\mathbf{382}\end{array}} $$

Final sample size with 10% non-response rate was **420**.

### Sampling technique

**Fig. 1:**
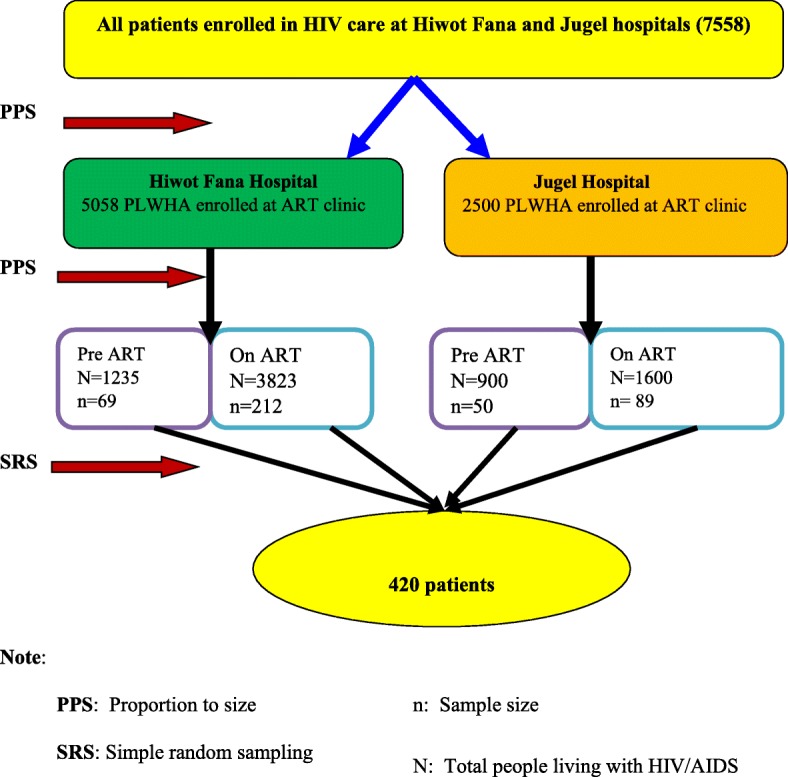
Sampling procedure of adult patients attending HIV follow up service in Harar town, Eastern Ethiopia, 2015

During our study period, there were 7558 registered HIV/AIDS patients enrolled in HIV services at both Hiwot Fana University specialized and Jugel governmental hospitals. Final sample size (420) was proportionally allocated to each hospital 281 from Hiwot Fana and 139 from Jugel hospital. At each hospital patients were stratified into pre-ART and ART based on their ART status. Then we selected eligible patients by simple random sampling technique based on their card number (Fig. 1).

### Data collection

We collected data through patient interview and review of medical records. Data regarding socio–demographics, CMDs, majority of clinical and psychosocial variables like (perceived HIV stigma, partner’s Sero-status, Children’s Sero status, death of either partner or family members due to HIV/AIDS, hospitalized in the last month, decreased level of functioning than usual, lost job due to HIV/AIDS, faced severe stress in the last 6 months, having family history of mental illness and having any comorbid chronic physical illness) and Substance-related variables (current drinking alcohol, chewing Khat, smoking cigarette, and using illicit substances) were retrieved by Interviewing patients using the adopted standardized questionnaire through face to face interview using interviewer-administered a standardized questionnaire. However, Respective medical Records like (HIV/AIDS stage, CD4 count and started ART) were obtained from patients’ medical record. We used Self Reporting Questionnaire (SRQ-20) to get data about CMDs. We used standardized questionnaire in interview-form instead of self-report because of the 16.2% (68) of our study participants were unable to read and write and they could not fill the questionnaire. The questionnaire was translated into local languages Afan Oromo and Amharic and validated to Ethiopian context. As common mental disorders (CMDs) refers to the set of signs and symptoms of non-psychotic depression, anxiety, and somatoform disorders, considering how challenging it can be to diagnose these disorders in primary care practice, the World Health Organization (WHO) developed the Self Reporting Questionnaire (SRQ-20), a screening instrument to identify suspected CMDs cases in primary care settings [21]. The SRQ-20 has 20 yes/no questions and it has been validated in Ethiopia, with high sensitivity (85.7%) and specificity (75.6%) [22]. Cut point of SRQ-20 is different in institution based and community based and usually cut-point of ≥7 is used in institution based [22]. Data collectors were 5 diploma nurses with previous data collection experience and supervisors were 3-graduate nurses and all of them were given one-days training prior to the data collection period. The study was conducted after approval obtained from the College of Health and Medical Sciences, Institutional Research Ethics Review Committee (IRERC) of Haramaya University. Written informed consent was obtained from the study participants after providing a clear explanation of the objective of the study. The collected data were kept confidential. Participants’ right to refuse and the chance to ask anything about the study was respected. The names of the participants were not written.

### Study Variables

#### Dependent variable

Status of common mental disorder (yes/no).

#### Independent Variables

**Socio-demographic variables** (age, sex, religion, ethnicity, marital status, occupational status, income, and educational status), **clinical and psychosocial variables** (HIV/AIDS stage, CD4 count, started ART, perceived HIV stigma, partner’s Sero-status, Children’s Sero status, death of either partner or family members due to HIV/AIDS, hospitalized in the last month, decreased level of functioning than usual, lost job due to HIV/AIDS, faced severe stress in the last 6 months, having family history of mental illness and having any comorbid chronic physical illness), **Substance-related variables** (current drinking alcohol, chewing Khat, smoking cigarette, and using illicit substances).

### Data processing and analysis

After checking for completeness data were entered into a computer using EpiData 3.1 then exported to SPSS for analysis. In statistical analysis we used categorical variables. Our dependent variable (CMD) is dichotomous which is categorized as yes (having CMD) or no (have no CMD). Since our dependent variable was dichotomous and we used cross-sectional study design, in order to explore the relationship between dependent variable and each independent variable we run a bivariate analysis using odds ratio (OR) of chi square. All independent variables that have a significant association with dependent variable (CMD) during bivariate analysis were adjusted together into multivariate analysis specifically binary logistic regression in order to control confounders. A *p* value of < 0.05 was considered as statistically significant association at 95% of confidence interval. We used Descriptive analysis to describe variables using frequencies, percentages, tables, and figures.

### Data quality control

To control the quality of data, we used the standard questionnaire in the Ethiopian context. The pretest was done at another Hospital and necessary adjustments were done before the actual data collection time. Data collectors collected data under the close supervision of trained supervisors. A one-day training about the objective of the study and other related issues was given for data collectors and supervisors.

### Operational Definition

**Common mental disorders:** Patients who scored ≥7 of SRQ-20 items were considered as having CMD [8]. **Current substance use:** Is defined as participants who had used substances at least once in the last one month prior to the study period [23].

### Ethical considerations

The College of Health and Medical Sciences Institutional Health Research Ethics Review Committee of Haramaya University had ethically approved the study. Permission from health facilities managers and written informed consent from patients were sought before administering questionnaires. We used anonymous questionnaires to keep patients’ confidentiality and the interview was conducted in a private room to assure the patients’ privacy. Patients with CMD were referred for professional care in the hospitals.

## Results

### Socio-demographic characteristics of respondents

From the total 420 patients, all were interviewed (providing response rate 100%); 26.0% of the respondents were males, and 71.0% were age less than 40 years. From the total participants, 53.6% were Oromo by ethnicity, 67.9% were Orthodox Christians, 37.6% were married and living together, 39.0% get a monthly pocket money of 500–999 Ethiopian birr, 44.3% educated primary school, and 17.4% were Government employee (Table 1).

**Table 1:** Socio-demographic characteristics of adult patients attending HIV follow up service in Harar town, eastern Ethiopia, 2015

Socio-demographic characteristics	Frequency (N)	Percent (%)
Sex		
Male	109	26.0
Female	311	74.0
Marital status		
Single	57	13.6
Married and living together	158	37.6
Separated	112	26.7
Divorced/widowed	93	22.1
Ethnicity		
Oromo	225	53.6
Amhara	118	28.1
Tigre	28	6.7
Guraghe	24	5.7
Harari	15	3.6
Others	10	2.4
Religion		
Muslim	94	22.4
Orthodox	285	67.9
Protestant	41	9.8
Age group in years		
< 40	298	71.0
40–60	114	27.1
> 60	8	1.9
Educational status		
Unable to read and write	68	16.2
Able to read and write	11	2.6
Primary school	186	44.3
Secondary school	120	28.6
Diploma	22	5.2
Degree	13	3.1
Occupational status		
House wife	51	12.1
Government employee	73	17.4
NGO employee	21	5.0
Private	48	11.4
Student	5	1.2
Daily laborer	102	24.3
Unemployed	56	13.3
Merchant	64	15.2
Income		
< 200	70	16.7
200–499	39	9.3
500–999	164	39.0
> =1000	147	35.0

*Others: Somali, Wolayita, and sidama*

### Prevalence of CMD among HIV/AIDS patients

The result revealed that among the 420 HIV/AIDS patients those were interviewed; Using SRQ-20 questionnaire (28.1%; 95% CI; 26.14, 30.06) of them had CMD.

### Factors associated with CMD among patients living with HIV/AIDS

The association between CMD and independent variables were determined using bivariate and multivariate (binary logistic regression) analyses. In the final model of multivariate logistic regression analysis HIV/AIDS stage and current drinking alcohol were found having statistically significant association with CMD. However, Socio-demographic variables like: Marital status and income were statistically significant in the binary logistic regression analysis but not significant in the final model of multivariate logistic regression analysis (Table 2).

**Table 2:** Binary logistic regression: Socio-demographic factors independently associated with CMD among adult patients attending HIV follow up service in Harar town, eastern Ethiopia, 2015

Variables	< 7 (no CMD)	> = 7 (CMD)	*p*-value	COR (95% CI)
Sex				
Male	82	27	Reference	Reference
Female	220	91	0.37	1.26[0.763–2.069]
Marital status				
Single	37	20	0.07	1.8[0.95–3.54]
Married & living together	122	36	Reference	Reference
Separated	73	39	0.03	1.8[1.06–3.1]
Divorced/widowed	70	23	0.73	1.1[0.6–2.03]
Ethnicity				
Oromo	171	54	Reference	Reference
Amhara	75	43	0.67	0.74[0.18–2.95]
Tigre	22	6	0.68	1.34[0.33–5.4]
Guraghe	18	6	0.58	0.64[0.13–3.24]
Harari	9	6	0.76	0.78[0.15–4.0]
Others	7	3	0.61	1.56[0.28–8.5]
Religion				
Muslim	61	33	0.51	1.31[0.59–2.89]
Orthodox	212	73	0.62	0.83[0.4–1.71]
Protestant	29	12	Reference	Reference
Age group in years				
< 40	208	90	0.14	1.5[0.9–2.4]
40–60	88	26	Reference	Reference
> 60	6	2	0.9	1.13[0.2–5.9]
Educational status				
Unable to read and write	43	25	0.15	3.2[0.66–15.61]
Able to read and write	8	3	0.48	2.06[0.28–15.36]
Primary school	137	49	0.39	1.9[0.42–9.19]
Secondary school	87	33	0.36	2.1[0.44–9.92]
Diploma	16	6	0.42	2.1[0.35–12.17]
Degree	11	2	Reference	Reference
Occupational status				
House wife	39	12	0.68	1.31[0.37–4.64]
Government employee	54	19	0.51	1.5[0.45–5.0]
NGO employee	17	4	Reference	Reference
Private	38	10	0.86	1.12[0.31–4.07]
Student	4	1	0.96	1.06[0.09–12.28]
Daily laborer	63	39	0.10	2.63[0.83–8.39]
Unemployed	39	17	0.33	1.85[0.54–6.33]
Merchant	48	16	0.58	1.42[0.42–4.83]
Income				
< 200	52	18	0.68	1.15[0.59–2.2]
200–499	27	12	0.33	1.48[0.68–3.2]
500–999	110	54	0.05	1.63[1.1–2.69]
> =1000	113	34	Reference	Reference

*Others: Somali, Wolayita, and sidama*

Likewise, Clinical, psychosocial and substance use factors like: perceived HIV stigma, lost job due to HIV/AIDS, having any comorbid chronic physical health problems, and current smoking cigarettes, chewing Khat and using illicit substances were again statistically significant in the binary logistic regression analysis but not significant in the final model (Table 3).

**Table 3:** Binary logistic regression: Clinical, psychosocial and substance use factors independently associated with CMD among adult patients attending HIV follow up service in Harar town, eastern Ethiopia, 2015

Variables	< 7 (no CMD)	> = 7 (CMD)	*p*-value	COR (95% CI)
CD4 count				
< =500cell/L	176	78	0.14	1.4[0.89–2.18]
>500cell/L	126	40	Reference	Reference
HIV/AIDS stage				
stage 1	187	54	Reference	Reference
stage 2	53	20	0.4	1.31[0.72–2.4]
stage 3	46	27	0.01	2.03[1.2–3.6]
stage 4	16	17	0.001	3.68[1.7–7.8]
Started taking ART				
Yes	291	109	0.09	0.46[0.19–1.14]
No	11	9	Reference	Reference
Perceived HIV stigma				
Yes	35	29	0.001	2.5[1.4–4.3]
No	267	89	Reference	Reference
Sero status of partner				
Positive	86	24	0.3	0.7[0.3–1.4]
Negative	45	18	Reference	Reference
Do not know	16	6	0.9	0.9[0.3–2.8]
No partner	155	70	0.7	1.13[0.6–2.1]
Children’s Sero status				
Yes	42	19	0.56	1.19[0.66–2.15]
No	258	98	Reference	Reference
Do not know	2	1	0.82	1.32[0.12–14.68]
Partner died because of HIV/AIDS				
Yes	61	21	0.6	0.86[0.49–1.48]
No	241	97	Reference	Reference
Family member died because of HIV/AIDS				
Yes	61	22	0.74	0.91[0.53–1.57]
No	240	95	Reference	Reference
Do not know	1	1	0.51	2.53[0.16–40.8]
Hospitalized in the last month				
Yes	16	9	0.37	1.48[0.63–3.44]
No	286	109	Reference	Reference
Level of functioning decreased than usual				
Yes	48	28	0.06	1.6[0.97–2.78]
No	251	89	Reference	Reference
Do not know	3	1	0.96	0.94[0.09–9.16]
Lost job due to HIV/AIDS				
Yes	43	26	0.05	1.7[1.1–2.93]
No	259	92	Reference	Reference
Family history of mental illness				
Yes	18	23	0.001	3.8[1.98–7.38]
No	284	95	Reference	Reference
Having any comorbid chronic physical health problem				
Yes	11	12	0.01	2.99[1.28–6.99]
No	291	106	Reference	Reference
Smoked cigarettes at least once during the last three months				
Yes	13	13	0.01	2.8[1.24–6.13]
No	289	105	Reference	Reference
Alcohol used at least once during the last three months				
Yes	30	39	0.001	4.48[2.61–7.66]
No	272	79	Reference	Reference
Chewed Khat at least once during the last three months				
Yes	48	37	0.001	2.417[1.471–3.971]
No	254	81	Reference	Reference
Used illicit substances at least once during the last three months				
Yes	17	16	0.008	2.63[1.28–5.39]
No	285	102	Reference	Reference

As revealed in final multivariate logistic regression analysis, clients who have stage 4 HIV/AIDS were 3.37 times more likely to have CMD than clients with stage 1 HIV/AIDS (AOR 3.37, 95% CI: 1.45, 7.83) and Patients who have family history of mental illness were 2.65 times more likely to develop CMD than clients who have no family history of mental illness (AOR 2.65, 95% CI: 1.26, 5.54). Those individuals currently drinking alcohol, were more than five times more likely to develop CMD than those who are currently not drinking (AOR 5.1, 95% CI: 2.04, 12.79) (Table 4).

**Table 4:** Multivariate logistic regression: Socio-demographic, Clinical, psychosocial and substance use factors independently associated with CMD among adult patients attending HIV follow up service in Harar town, eastern Ethiopia, 2015

Variables	< 7 (no CMD)	> = 7 (CMD)	*p*-value	AOR (95% CI)
Marital status				
Single	37	20	0.07	2.01[0.94–4.30]
Married and living together	122	36	Reference	Reference
Separated	73	39	0.3	1.4[0.75–2.58]
Divorced/widowed	70	23	0.98	0.9[0.49–1.98]
Income				
< 200	52	18	0.75	0.88[0.41–1.88]
200–499	27	12	0.98	1.01[0.41–2.45]
500–999	110	54	1.18	1.5[0.84–2.59]
> = 1000	113	34	Reference	Reference
HIV/AIDS stage				
stage 1	187	54	Reference	Reference
stage 2	53	20	0.86	0.94[0.48–1.84]
stage 3	46	27	0.28	1.42[0.75–2.69]
stage 4	16	17	0.005	3.37[1.45–7.83]
Perceived HIV stigma				
Yes	35	29	0.09	1.78[0.92–3.43]
No	267	89	Reference	Reference
Lost job due to HIV/AIDS				
Yes	43	26	0.17	1.57[0.83–2.98]
No	259	92	Reference	Reference
Family history of mental illness				
Yes	18	23	0.01	2.65[1.26–5.54]
No	284	95	Reference	Reference
Having any chronic physical health problem				
Yes	11	12	0.22	1.85[0.69–4.95]
No	291	106	Reference	Reference
Smoked cigarettes at least once during the last three months				
Yes	13	13	0.94	1.05[0.34–3.19]
No	289	105	Reference	Reference
Alcohol used at least once during the last three months				
Yes	30	39	0.001	5.1[2.04–12.79]
No	272	79	Reference	Reference
Chewed Khat at least once during the last three months				
Yes	48	37	0.21	0.56[0.23–1.38]
No	254	81	Reference	Reference
Used illicit substances at least once during the last three months				
Yes	17	16	0.086	2.27[0.99–5.98]
No	285	102	Reference	Reference

## Discussion

This study showed that the overall prevalence rate of common mental disorder among HIV/AIDS patients is 28.1% with the range between 26.14 and 30.06%. Associated factors identified in the final model of this study were stage of HIV/AIDS, Family history of mental illness and current drinking alcohol.

In the current study the prevalence of common mental disorder among people living with HIV/AIDS is closer to the finding in south west regional hospitals of Cameroon, which shows 26.7% of HIV/AIDS patients on HAART have depression [24]. On the other hand, the prevalence is greater than that found in Tanzania and South Africa [25, 26] in which the prevalence was 15.5 and 14.2% respectively. This variation could be due to different possible reasons like different method used to assess the condition. The study in Tanzania conducted using ICD-10 common mental health diagnosis and in South Africa they applied DSM diagnoses. But in the current study we applied SRQ-20 standardized questionnaire. The present study result is lower than the studies done in Zimbabwe and Indian setting [27, 28] in which the findings were 67.9 and 58.75% respectively. This difference could be the slight difference of study population nature like in the study conducted in Zimbabwe 92% of the participants were on HAART, which indicates that they could have severe illness. As the severity of HIV/AIDS increased the patients could develop CMD more [27]. But in the current study only 71.7% were on HAART and the left were pre-ART. The other possible reason could be in the present study majority of the participants were young age as 71.0% of them were at age less than 40 years. Study on common mental disorder among general population showed that older age is significantly associated with higher prevalence of common mental disorder [29].

Our study finding shown that patients who have stage 4 HIV/AIDS were more likely to develop common mental disorder (AOR 3.37, 95% CI: 1.45, 7.83) and it is in line with the results of studies done in India and Ethiopia [27, 30]. Patients those are currently drinking alcohol are also more likely to develop common mental disorder (AOR 5.1, 95% CI: 2.04, 12.79) and this result is supported by study conducted on common mental disorder among general population in Ethiopia [29]. As more alcohol enters the bloodstream, the areas of the brain associated with emotions and movement are affected, often resulting in exaggerated states of emotion (anger, withdrawal, depression or aggressiveness) and uncoordinated muscle movements [31]. Also, our study revealed that patients who had family history of mental illness were more likely to have common mental disorder as compared to patients who have no family history of mental illness (AOR 2.65, 95% CI: 1.26, 5.54).

### Limitations of the study

The study was hospital based and some patients with severe common mental disorder might unlikely to be available in the hospital due to their severity of common mental disorder during data collection period. This could influence the prevalence of common mental disorder and it might not be generalized to the total population of people living with HIV/AIDS in the region. Also, our study was cross-sectional and it cannot show the cause-effect relationship between common mental disorder and independent variables.

## Conclusions

In this study the prevalence of CMD is relatively high. HIV/AIDS stage, having family history of mental illness and current drinking alcohol were the identified factors those have significant association with common mental disorder among patients living with HIV/AIDS. The study finding will provides information to form rational foundation for prevention and planning to bring change in contributing factors for developing CMD among patients living with HIV/AIDS and also will be base line information for further study. The study recommended that it is important to control the progression of HIV like early detecting and treating opportunistic infections.

## Abbreviations

AIDS: Acquired Immune Deficiency Syndrome
AOR: Adjusted Odds Ratio
ART: Antiretroviral Therapy
CMD: Common Mental Disorders
COR: Crude Odds Ratio
FGAE: Family Guidance Association of Ethiopia
HAART: Highly Active Antiretroviral Therapy
HIV: Human Immune Virus
IRERC: Institutional Research Ethics Review Committee
LAMIC: Low- and Middle-Income Countries
MDD: Major Depressive Disorder
PLWHA: People Living with HIV/AIDS
QOL: Quality of Life
SRQ: Self-Reported Questionnaire
STI: Sexually Transmitted Infections
WHO: World Health Organization
